# Predictive metacognition: a neuro-computational framework for self-monitoring in large language models

**DOI:** 10.1038/s41598-026-54840-2

**Published:** 2026-05-26

**Authors:** Wei Luo, Hunkoog Jho

**Affiliations:** 1 School of Information Technology, Deakin University, 221 Burwood Highway, Burwood, Melbourne, VIC 3125 Australia; 2 Institute of Knowledge Integration & Design, Dankook University, 152, Jukjeon-ro, Suji-gu, Yongin, Gyeonggi-do 16890 Republic of Korea

**Keywords:** Metacognition, Cognitive architecture, Predictive processing, Confidence calibration, Self-monitoring, Neural computation, Computational biology and bioinformatics, Mathematics and computing, Neuroscience, Psychology, Psychology

## Abstract

Large Language Models demonstrate remarkable capabilities but suffer from critical metacognitive deficits, manifesting as overconfidence and hallucination, which severely limit their deployment in high-stakes applications. We introduce Predictive Metacognition, a neurobiologically-inspired framework that integrates principles of predictive processing and anterior cingulate cortex monitoring into transformer architectures. Our approach implements Error-Driven Learning and Dual-Process Monitoring through specialised fine-tuning that trains models to simultaneously generate responses and assess their own performance reliability. We fine-tuned Llama-3-8B-Instruct and Phi-3-Mini-4k-Instruct using LoRA (rank=8, *α =16*) on 4,000 strategically constructed examples spanning varying confidence levels. Comprehensive evaluation against state-of-the-art baselines, including GPT-4o and Claude-3.5-Sonnet, revealed statistically significant improvements in confidence calibration. Our metacognitive models achieved substantial reductions in Brier Score (11.6% and 17.2% respectively) and Expected Calibration Error (*p < 0.023*, Cohen’s *d = 1.456*). Critically, these improvements generalised robustly to out-of-domain tasks while maintaining competitive task accuracy. This work establishes a computationally tractable implementation of biologically-inspired metacognitive architecture for large language models, offering a principled pathway towards AI systems capable of reliable intrinsic self-monitoring that can more accurately assess their own knowledge boundaries and express appropriate uncertainty.

## Introduction

The proliferation of Large Language Models (LLMs) represents a pivotal advancement in artificial intelligence (AI), demonstrating remarkable capabilities in text generation, summarisation, and complex reasoning^1,2^. These models have become integral to a wide array of applications, reshaping industries and scientific inquiry. However, their operational utility is persistently undermined by a fundamental and critical flaw: a profound lack of metacognitive capabilities. This deficit manifests as a tendency towards fabricating information, a phenomenon widely termed “hallucination”^3^, and an unwarranted overconfidence in their own outputs, irrespective of their factual accuracy. LLMs generate responses based on statistical patterns learned from vast datasets, but they do not possess an intrinsic mechanism to differentiate between verified knowledge and plausible-sounding falsehoods. This inability to reliably assess their own knowledge boundaries or the veracity of their statements makes their behaviour brittle and poses significant risks, limiting their deployment in high-stakes domains where trustworthiness is paramount.

The core of this reliability crisis can be traced to the absence of what cognitive science defines as metacognition^4^. This gap is actively being investigated, with recent studies indicating that even when prompted, models’ verbalised statements of confidence often fail to align with their actual performance^5^. This highlights a fundamental lack of capacity to monitor, evaluate, and regulate one’s own cognitive processes. For humans, metacognition is crucial for robust learning and effective decision-making; it is the faculty that allows us to realise when we are uncertain, to double-check a fact, or to adjust a problem-solving strategy that is failing. Current LLMs largely lack this self-aware layer. Efforts to mitigate this issue have predominantly focused on reactive or supplementary solutions. For instance, Retrieval-Augmented Generation (RAG) bolts on external knowledge bases to ground model outputs in factual data^6^, while prompting strategies like Chain-of-Thought (CoT) aim to make the model’s reasoning process more transparent and thus more criticisable^7^. Although valuable, these approaches, along with recent work on iterative self-refinement where models correct their own output after generation^8^, are primarily external augmentations or post-hoc verifications. They do not endow the model with an innate, proactive ability for self-monitoring during the initial generation process.

To fundamentally address this gap, we propose a new framework, Predictive Metacognition, which integrates principles of neurobiological self-monitoring directly into the model’s architecture. Our approach is inspired by two foundational concepts in human cognitive neuroscience: predictive processing and the function of the anterior cingulate cortex (ACC). Predictive processing theories posit that the brain is a sophisticated prediction machine, constantly generating models of the world and updating them based on the mismatch between prediction and sensory input, or prediction error^9,10^. Concurrently, the ACC is understood to be a critical neural substrate for performance monitoring and cognitive control, exhibiting heightened activity during conflict detection and error signalling^11^. Importantly, the ACC’s function extends beyond simple error detection; it is crucial for signalling the need for cognitive adjustment when conflicts or errors are detected, a principle we aim to model computationally^12^. It is important to note, however, that the ACC comprises functionally distinct subregions: dorsal ACC is broadly implicated in cognitive control and conflict monitoring; rostral ACC in emotional regulation; subgenual ACC in autonomic functions and mood control^13^; while pregenual ACC (pgACC) has been more specifically associated with encoding uncertainty signals akin to Pearce-Hall associability—that is, running averages of absolute prediction errors—as demonstrated in studies of relief learning and adaptive pain control^14,15^. Recent computational work has further shown that such pgACC-encoded uncertainty signals may serve to arbitrate between reactive Pavlovian and deliberative instrumental behavioural systems^16^. Our framework draws on this more nuanced functional role of ACC subregions in uncertainty-driven self-monitoring, rather than treating the ACC as a monolithic error-detection module. By synthesising these principles, our framework reframes the task of an LLM. Instead of merely predicting the next token in a sequence, the model is trained to simultaneously predict its own performance and estimate the likelihood of its response being correct. This shifts the paradigm from reactive, external correction to proactive, internal self-assessment, enabling the model to learn an internal model of its own uncertainty.

This paper makes several key contributions toward more reliable and self-regulating AI. First, we introduce the Predictive Metacognition framework, which integrates neuroscientific principles of self-monitoring into LLM architecture. Second, we present a practical and efficient fine-tuning methodology to implement this framework, enhancing the model’s self-assessment capabilities. Third, through comprehensive evaluation against state-of-the-art baselines, we provide rigorous empirical evidence that our approach significantly improves confidence calibration and other reliability metrics. These contributions are validated through detailed analyses and ablation studies, demonstrating the effectiveness of our proposed components.

## The framework of predictive metacognition

While the capabilities of Large Language Models are remarkable, their reliability is undermined by a key limitation: a lack of intrinsic metacognition. Although modern transformer architectures contain implicit signals related to confidence, such as attention entropy and cross-head consensus, they lack explicit mechanisms to utilize this information for self-monitoring. This deficiency often leads to overconfidence and “hallucination”, hindering their use in high-stakes domains. To address this gap, we must move beyond post-hoc corrections and instead build proactive self-assessment directly into the model’s architecture.

Modern transformer architectures contain numerous implicit signals that correlate with output reliability and confidence, though these signals are neither explicitly utilised nor accessible for metacognitive control. Research has demonstrated that attention entropy–the concentration or distribution of attention weights across input tokens–serves as a reliable confidence indicator across various language tasks^17,18^. When attention weights are sharply focused on specific input tokens, indicating low entropy, models typically produce more accurate and reliable outputs. Conversely, when attention is diffusely distributed across many tokens, reflecting high entropy, outputs tend to be less reliable and more prone to errors. This pattern mirrors findings from human neuroscience, where focused attention correlates with increased confidence and improved performance^19^.

Cross-head consensus provides another implicit uncertainty signal that current systems fail to exploit effectively. Different attention heads within the same layer often focus on distinct aspects of input, essentially providing multiple perspectives on information relevance^20,21^. When heads demonstrate high consensus–focusing on similar token sets–models typically produce more reliable outputs. When heads disagree significantly about attention allocation, outputs tend to be less reliable. Despite the presence of these implicit metacognitive signals, current architectures lack explicit mechanisms to utilise this information for error detection, strategy adjustment, or confidence communication.

The most profound limitation constraining metacognitive development in current LLMs stems from their token-by-token processing paradigm. This sequential approach, while computationally efficient and enabling impressive scaling, prevents the global planning and retrospective evaluation that characterises human metacognition^21,22^. Token-level processing creates conceptual fragmentation where important ideas and relationships are distributed across multiple discrete units rather than being represented as unified cognitive objects. The absence of global coherence monitoring represents perhaps the most serious metacognitive deficit in current systems. Because generation proceeds left-to-right without global optimisation, models cannot detect contradictions between early and late portions of their outputs.

Recent efforts to enhance LLM metacognitive capabilities have pursued several promising directions, yet these approaches remain constrained by fundamental architectural limitations and their reactive rather than proactive nature. Post-hoc confidence calibration represents the most widespread approach to improving metacognitive performance. Temperature scaling adjusts SoftMax temperatures to improve probability calibration^23^, while ensemble methods combine predictions from multiple models to estimate uncertainty^24^, and conformal prediction methods provide distribution-free uncertainty quantification by constructing prediction sets with guaranteed coverage^25^. Critically, these post-hoc methods operate at inference time on frozen models and do not modify the learning objective–representing a fundamentally different level of intervention from our training-time approach. Although these techniques can improve calibration metrics, they operate at surface levels without addressing underlying metacognitive deficits. Chain-of-Thought prompting encourages models to generate step-by-step reasoning, potentially improving performance on complex problems^7,26^. However, CoT reasoning remains fundamentally sequential and lacks ability to revise earlier steps based on later insights.

To fundamentally address this gap and move beyond architectures that are powerful yet brittle, we turn to biological intelligence, which offers a proven blueprint for robust and adaptive self-monitoring honed over millions of years of evolution. Our framework is predicated on the theory of predictive processing, which posits that the brain is not a passive engine for stimulus processing, but an active, inferential organ that constantly generates predictions about the causes of its sensory input^9,10^. In this view, perception and cognition are processes of hypothesis testing. The brain constructs a generative model of its environment to anticipate sensory signals, and this framework has recently been extended to provide sophisticated computational accounts of how subjective confidence itself may arise from the geometry of these predictive models^27^.

The crucial element of this process is the signal of prediction error: the mismatch between the brain’s predictions and the actual sensory evidence. This error signal is not a failure but the primary driver of learning and belief updating, compelling the brain to revise its internal model to minimise future errors and better account for the world. This principle of error-based model updating finds a compelling neuroanatomical substrate in the anterior cingulate cortex (ACC). The ACC is widely recognised as a central hub for performance monitoring and cognitive control, exhibiting heightened activity when detecting conflicts between competing responses, unexpected outcomes, or errors in judgement^11^. As noted above, the ACC comprises functionally distinct subregions, with pgACC playing a particularly relevant role in encoding uncertainty signals that drive self-monitoring and behavioural adjustment^14,15^. Importantly, the ACC’s function extends beyond simple error detection; it is crucial for signalling the need for cognitive adjustment when conflicts or errors are detected, a principle we aim to model computationally^12^. Neuroscientific research provides compelling evidence for this architectural specialisation. The human brain contains distinct prefrontal regions dedicated to monitoring, controlling, and integrating metacognitive information^28^. The rostro-lateral prefrontal cortex (rlPFC) serves as a domain-general metacognitive hub, while the anterior cingulate cortex (ACC) specialises in error monitoring and conflict detection. Lesions to these regions produce specific metacognitive deficits while leaving first-order cognitive abilities largely intact^29^. Recent neuroscientific research has identified specific brain regions and mechanisms responsible for metacognitive processing across different sensory modalities. Obleser introduced the “meta-listening” framework, demonstrating how alpha oscillatory activity and cortical excitation-inhibition balance shape metacognitive outcomes in auditory processing, revealing that metacognitive capabilities extend beyond visual and memory domains to encompass fundamental aspects of sensory processing^30^. While the ACC provides a key substrate for error-based metacognitive monitoring, confidence representations are not confined to this region. Empirical evidence for domain-general confidence representations has been demonstrated in animal studies, with Masset et al. identifying neurons in rat orbitofrontal cortex that encode statistical decision confidence irrespective of sensory modality^31^. This biological evidence suggests that effective AI metacognition may similarly require dedicated architectural components operating in parallel with primary processing systems.

**Fig. 1:**
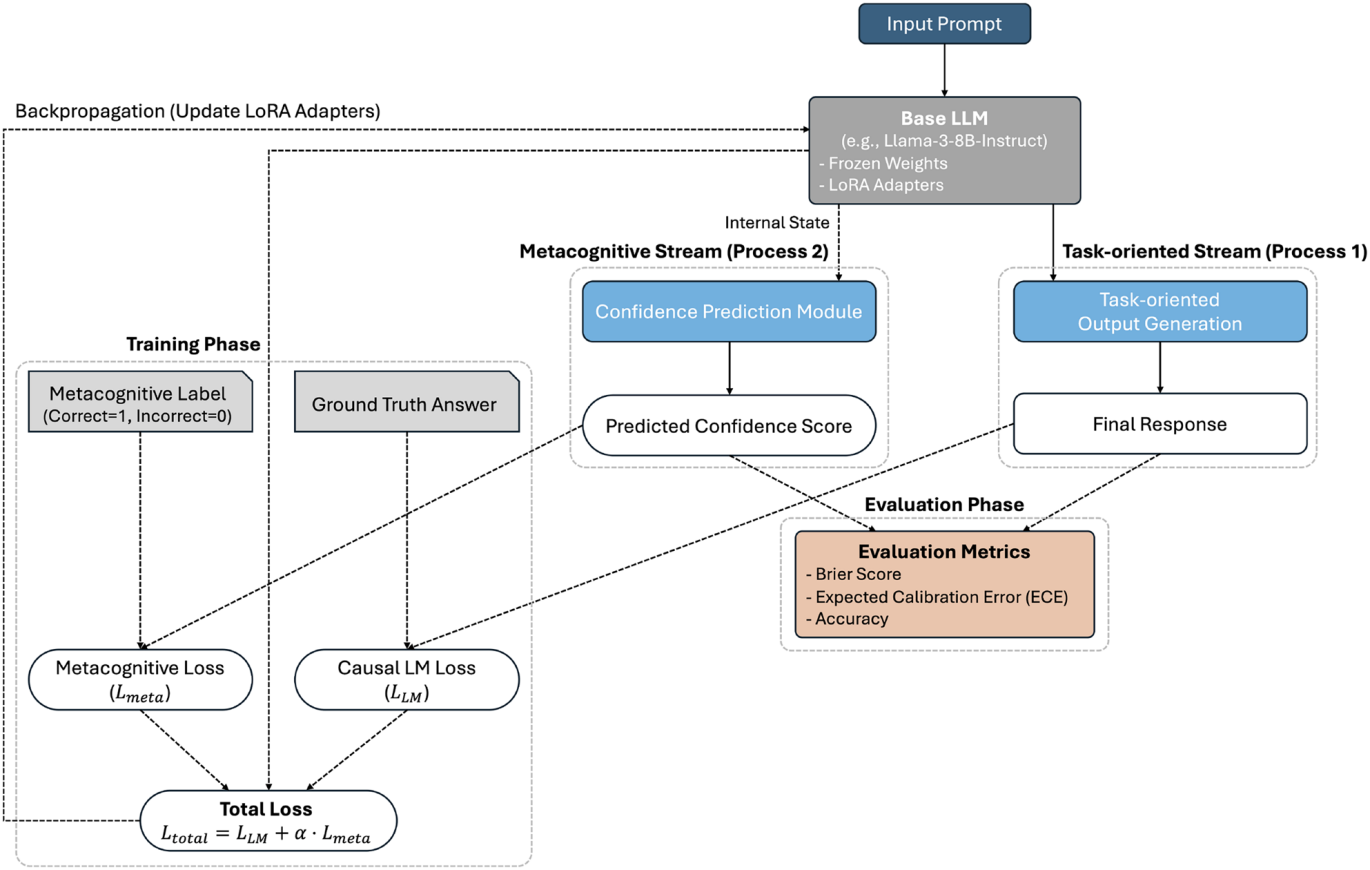
Architectural diagram of the predictive metacognition framework.

Predictive Metacognition operationalises these neurobiological concepts by re-framing the fundamental learning objective of an LLM. A central feature of this operationalisation is the conceptual separation of the model’s cognitive labour into two distinct, parallel streams: a primary task-oriented process responsible for generating content, and a secondary metacognitive process dedicated to monitoring the quality and certainty of that generation. The overall architecture of this framework, which separates the training and evaluation phases, is visualized in Fig 1. We realise this architecture through four core principles designed to be integrated within a Transformer-based model:

- *Hierarchical prediction*: The model learns to make predictions across multiple levels of abstraction simultaneously. This extends beyond low-level token prediction to encompass higher-order assessments, such as the logical consistency of an argument or the overall probability of success on the given task. This mirrors the hierarchical organisation of the cerebral cortex, where early stages handle basic features while later stages integrate information into coherent interpretations.
- *Error-driven learning*: The central learning signal is the metacognitive prediction error–the discrepancy between the model’s predicted confidence in its own output and the actual, ground-truth outcome. By explicitly training the model to minimise this error, it learns to generate more accurately calibrated self-assessments. This reflects the principle that confidence emerges from statistical computations over neural population activity rather than requiring separate metacognitive systems, as proposed by Meyniel et al.^32^ who argued that “uncertainty” in neural computation and “confidence” in metacognitive research reflect different manifestations of the same underlying Bayesian probability computation mechanism. The role of prior expectations in shaping metacognitive judgements has been demonstrated through Bayesian frameworks, with Boldt et al.^33^ showing that prior probabilistic expectations facilitate metacognitive accuracy in perceptual decision-making, supporting the hypothesis that top-down influences modulate both task performance and confidence estimation.
- *Attention-based confidence estimation*: The model’s confidence is not treated as a single, post-hoc judgement. Instead, it is dynamically estimated by learning to interpret patterns within its own internal states. The model learns which attention head activations and token representations are most indicative of a correct or incorrect reasoning path, allowing it to ground its self-assessment in the mechanics of its own computation. This leverages the mathematical relationship of attention entropy $H(A) = -\sum _i a_i \log (a_i )$, where $a_i$ represents the attention weight for token i, with values approaching zero indicating highly focused attention patterns associated with high confidence.
- *Dual-process monitoring*: This principle formalises the parallel operation of the two streams introduced above. It ensures that the task-oriented stream (Process 1) and the metacognitive stream (Process 2) are trained and operate concurrently, enabling real-time monitoring of cognitive processes rather than post-hoc evaluation. This approach aligns with theoretical frameworks distinguishing between different types of metacognitive processing, as Shea et al.^34^ proposed that metacognitive representations can become available for verbal report and communication, enabling more sophisticated forms of collaborative cognition through their supra-personal cognitive control framework. The development of computational models of metacognition has been guided by the need to bridge neuroscientific findings with practical applications. Fleur et al.^35^ reviewed the convergence between neuroscientific and educational approaches to metacognition, identifying key entry points for synthesis and revealing opportunities for developing AI systems that can both implement neurobiologically-plausible metacognitive mechanisms and support human learning. Recent theoretical developments have emphasised the need to balance construct breadth with measurement rigour, as Katyal and Fleming^36^ argued that restoring construct breadth while maintaining rigorous measurement standards could lead to more comprehensive understanding of metacognitive phenomena with implications for developing AI systems that possess more robust metacognitive capabilities. Cross-cultural research has revealed that metacognitive capabilities are modulated by cultural values and social contexts, with Ordin et al.^37^ demonstrating that metacognitive efficiency varies systematically across societies, highlighting the importance of considering contextual factors when designing AI metacognitive systems. Research on metacognitive efficiency and bias has revealed complex relationships between different aspects of self-assessment, with Shekhar and Rahnev^38^ finding robust positive correlations between metacognitive efficiency and metacognitive bias, suggesting that confidence calibration and discrimination accuracy may not be independent capabilities in AI system design.

To implement this framework, these principles must be formalised into a computable learning objective. The Dual-Process Monitoring principle, with its explicit separation of Process 1 and Process 2, naturally lends itself to a composite loss function that optimises both streams of processing. The first component is the standard causal language modelling loss, $L_{LM}$, which trains the task-oriented stream (Process 1) to generate coherent and contextually appropriate text via next-token prediction. It is typically calculated using the Cross-Entropy loss:

1 $$\begin{aligned} L_{\textrm{LM}} = -\sum _i \log P\bigl (t_i \mid t_{<i}\,\text {context}\bigr ) \end{aligned}$$

The second, and novel, component is the metacognitive loss, $L_{meta}$, which trains the self-monitoring stream (Process 2). This loss function quantifies the metacognitive prediction error. It penalises the divergence between the model’s expressed confidence prediction, $\hat{c}$ (a scalar value between 0 and 1), and the ground-truth correctness of its response, $y_{meta}$ (where 1 signifies a correct response and 0 an incorrect one). Critically, $y_{meta}$ is defined at the level of the entire response rather than individual tokens: it is assigned a value of 1 if the model’s complete generated response is judged correct against the ground-truth answer, and 0 otherwise. This response-level supervision ensures that the metacognitive signal reflects holistic output quality rather than token-level prediction accuracy. For this, we employ the Binary Cross-Entropy (BCE) loss:

2 $$\begin{aligned} L_{\textrm{meta}} = -\bigl [y_{\textrm{meta}}\log (\hat{c}) + (1 - y_{\textrm{meta}})\log \bigl (1 - \hat{c}\bigr )\bigr ] \end{aligned}$$

The final training objective combines these two components into a single function, $L_{total}$. This allows for the end-to-end optimisation of both task performance and self-assessment capability. The two losses are balanced by a hyperparameter, *α*, which controls the relative weight of the metacognitive training objective. This parameter corresponds directly to the meta_loss_alpha argument in our implementation.

3 $$\begin{aligned} L_{total} = L_{LM} + \alpha \cdot L_{meta} \end{aligned}$$

This framework represents a fundamental shift from reactive, external correction to proactive, internal self-assessment, enabling the model to learn an internal model of its own uncertainty. Unlike surface-level modifications to existing systems, this approach addresses the architectural constraints that prevent genuine metacognitive awareness by introducing dedicated computational mechanisms for real-time self-monitoring. The biological inspiration ensures that the framework is grounded in proven principles of robust cognitive control, while the mathematical formalisation provides a clear and reproducible implementation pathway.

The development of genuinely metacognitive AI systems therefore requires moving beyond incremental improvements to existing architectures towards fundamental innovations inspired by the computational principles underlying biological metacognition. Such systems integrate predictive self-monitoring, hierarchical cognitive control, and real-time strategy adjustment as core architectural features rather than post-hoc additions. Having established the theoretical framework and its corresponding learning objective, the following chapter will detail the practical methodology used to implement, train, and evaluate models based on these principles.

## Experimental methodology

To empirically test the principles of Predictive Metacognition laid out in this study, we designed a rigorous and multi-faceted experimental methodology. The central hypothesis of this research is that fine-tuning a Large Language Model using our neuro-inspired framework will significantly improve its self-assessment capabilities, and critically, that this learned metacognitive skill will generalise to new, unseen domains. This chapter details the deliberate choices made in our experimental design to validate this hypothesis.

Our experimental design is built upon three key comparison strategies. First, to test the core effectiveness of our method, we directly compare our fine-tuned models against their original base versions. Second, for our ablation study, we compare our models against prompt-engineered baselines to isolate the unique contribution of our training methodology beyond what can be achieved with simple prompting. Finally, to situate our work within the broader landscape, we perform SOTA (State-of-the-Art) benchmarking against leading proprietary models, assessing the practical relevance and competitiveness of our approach.

The models central to this study reflect this multi-tiered comparison strategy. We selected two powerful open-foundation models as the basis for our work: Meta’s Llama-3-8B-Instruct and Microsoft’s Phi-3-Mini-4k-Instruct. This selection was deliberate and reflects a core motivation of this work: to demonstrate that targeted metacognitive fine-tuning of relatively compact, open-source models is sufficient to address confidence miscalibration–offering a computationally accessible alternative to reliance on large proprietary systems. As open-weight models, Llama-3-8B-Instruct and Phi-3-Mini-4k-Instruct permit direct modification of their parameters, which is a prerequisite for implementing our intrinsic fine-tuning approach. By applying our fine-tuning process, we created the two proposed models at the core of our investigation: Finetuned Llama-3-8B-Instruct (Metacognitive-Llama) and Finetuned Phi-3-Mini-4k-Instruct (Metacognitive-Phi). These were evaluated against a comprehensive suite of baselines, including their original, unmodified versions (Base-Llama-3-8B-Instruct, Base-Phi-3-Mini-4k-Instruct), their prompt-engineered counterparts (Prompted-Llama-3-8B-Instruct, Prompted-Phi-3-Mini-4k-Instruct), and leading SOTA closed models: OpenAI’s GPT-4o and Anthropic’s Claude-3.5-Sonnet. The remaining open-source models–Mistral-7B, Gemma-7B, and Qwen2-7B–serve as additional baselines to broaden the comparison landscape, while GPT-4o and Claude-3.5-Sonnet represent closed proprietary systems whose weights are inaccessible and therefore cannot serve as fine-tuning targets within this research context.

**Fig. 2:**
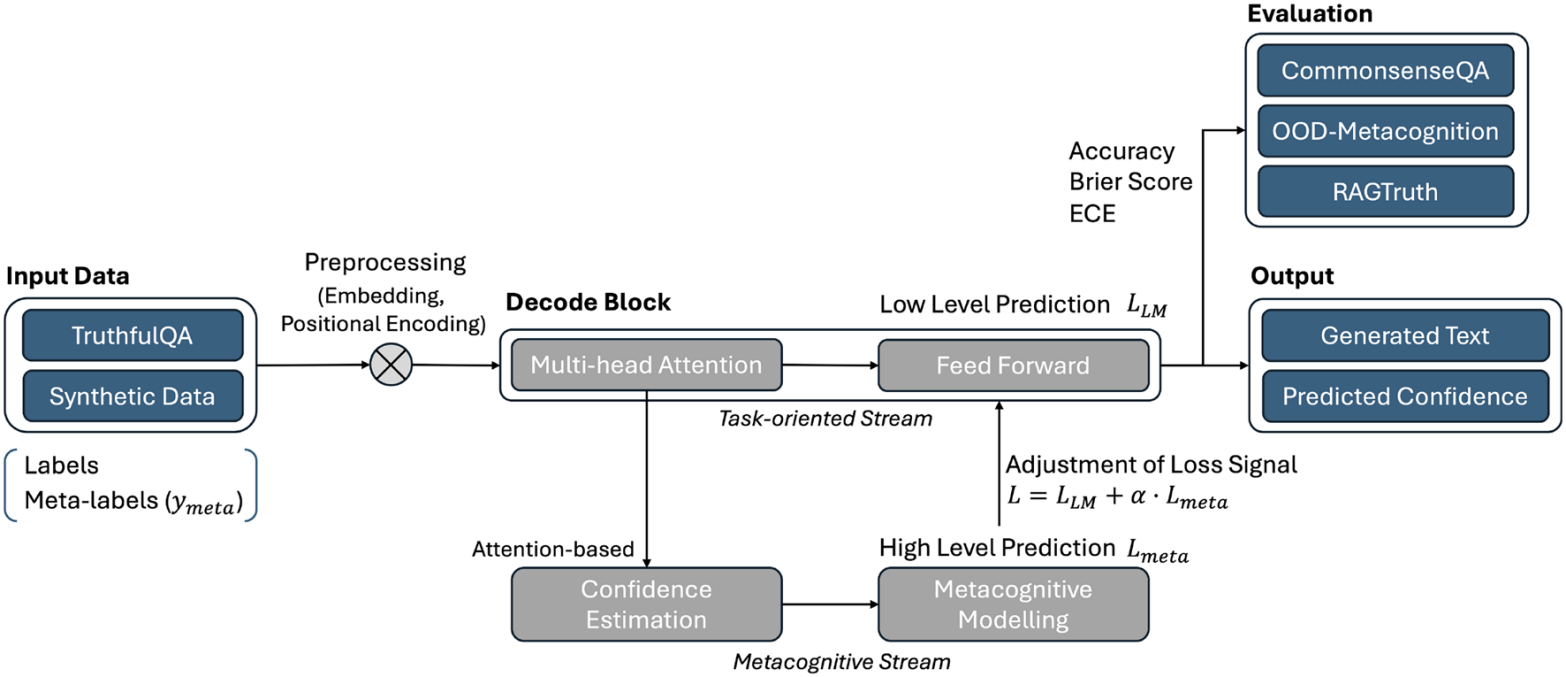
Detailed end-to-end pipeline of the predictive metacognition framework.

The architectural implementation of our framework is visualised in both Figs. 1,2. This diagram illustrates how a single input prompt initiates a dual-stream process within the model. The Task-Oriented Stream (Process 1) proceeds with the conventional task of generating a textual response. In parallel, the Metacognitive Stream (Process 2) concurrently monitors the internal computational states of Process 1 to produce a predictive confidence score assessing the likely correctness of the final output. This dual-output system forms the basis of our implementation. To realise this architecture efficiently, we employed Low-Rank Adaptation (LoRA), a parameter-efficient fine-tuning (PEFT) technique^39^. The training process was designed to optimise a composite loss function ($L_{total}=L_{LM}+\alpha \cdot L_{meta}$), which simultaneously governs both the task-oriented and metacognitive processing streams.

Figure 2 illustrates the entire process, starting from the input of TruthfulQA and Synthetic Data which provide meta-labels ($y_{meta}$). As illustrated, $y_{meta}$ is a response-level binary label: it is assigned 1 if the model’s complete generated response is correct against the ground-truth answer, and 0 otherwise, ensuring that the metacognitive supervision signal reflects holistic output quality rather than token-level prediction accuracy. The data is preprocessed and enters a Decode Block, where the Task-oriented Stream generates text (leading to $L_{LM}$) and the Metacognitive Stream uses attention-based estimation to predict confidence (leading to $L_{meta}$). The composite loss L updates the model’s trainable parameters. Finally, the model’s Output (Generated Text and Predicted Confidence) is benchmarked against evaluation datasets using metrics including Accuracy, Brier Score, and ECE.

To realise this architecture efficiently, we employed Low-Rank Adaptation (LoRA), a parameter-efficient fine-tuning technique^39^. Our LoRA configuration consisted of rank (r) = 8, alpha = 16, targeting the query, value, and output projection modules [“q_proj”, “v_proj”, “o_proj”], with a dropout rate of 0.1. This configuration enabled fine-tuning of approximately 0.52% of the total model parameters while maintaining computational efficiency.

Our implementation of the Metacognitive Stream (Process 2) employs a two-stage approach for computational efficiency. First, an initial confidence score is estimated directly from the model’s internal states using attention entropy ($H(A) = -\sum _i a_i \log (a_i)$), a computationally inexpensive method. Second, if this initial confidence falls below a predefined threshold (e.g., 0.3), a more resource-intensive prediction head (a small neural network) is engaged to generate a refined confidence assessment. The threshold value of 0.3 was determined empirically through systematic evaluation on the validation set, where it yielded the most favourable trade-off between computational overhead and calibration performance across both model architectures. While this value proved effective in the present study, we acknowledge that the optimal threshold may vary across different model families and tasks; a comprehensive sensitivity analysis of this hyperparameter remains an avenue for future work. This conditional activation allows the model to balance speed and accuracy in its self-monitoring. In Fig. 2, this two-stage process is represented by the ’Attention-based Confidence’ and ’Metacognitive Modeling’ components within the Metacognitive Stream.

Training was conducted with carefully optimised hyperparameters: learning rate of 3e-4, 3 epochs, per-device batch size of 2 with gradient accumulation steps of 8 (effective batch size: 16), maximum sequence length of 256 tokens, and metacognitive loss weight (*α*) of 0.1. We employed AdamW optimiser with cosine learning rate scheduling, 100 warmup steps, weight decay of 0.01, and gradient clipping at 1.0 to ensure training stability.

The composite loss function was implemented as $L_{total}=L_{LM}+\alpha \cdot L_{meta}$ where $L_{LM}$ represents the standard causal language modelling loss using CrossEntropyLoss with ignore_index=-100, and $L_{meta}$ employs BCELoss (or BCEWithLogitsLoss for numerical stability) between the model’s predicted confidence and the ground-truth labels, consistent with our framework’s definition.

Our methodology for data selection was highly strategic, designed to provide comprehensive training signals for metacognitive learning. The metacognitive fine-tuning was conducted on a carefully constructed dataset of 4,000 examples specifically designed to teach the model to differentiate between valid and flawed reasoning patterns across varying confidence levels. The training dataset comprised multiple components designed to capture the full spectrum of metacognitive behaviour. The foundation was built upon TruthfulQA validation set examples, supplemented by synthetic question-answer pairs with systematically varied confidence levels. High confidence examples (*\ge 0.7*) consisted of factual knowledge questions with definitive answers, such as scientific facts and mathematical calculations. Medium confidence examples (*0.3-0.7*) included contextual or opinion-based questions where certainty is inherently limited. Low confidence examples (*<0.3*) featured uncertain predictions, future forecasts, and explicit uncertainty disclaimers. Examples of our training data structure include high-confidence instances like “Question: What is the chemical formula for water? Answer: The chemical formula for water is H2O”(confidence: 0.95), medium-confidence instances such as “Question: What causes climate change? Answer: Climate change is primarily caused by human activities, particularly greenhouse gas emissions” (confidence: 0.7), and low-confidence instances like “Question: What will the stock market do next year? Answer: I cannot predict future stock market movements with certainty” (confidence: 0.1). This stratified approach ensured balanced representation across the confidence spectrum, with the final dataset maintaining a 90%/10% train-validation split, providing the foundational signal for the model to build an internal representation of appropriate confidence calibration. The use of 4,000 training examples was a deliberate design choice to demonstrate the sample efficiency of our framework. Prior work has shown that parameter-efficient fine-tuning methods such as LoRA can achieve significant behavioural shifts with limited data by updating only a small fraction of model parameters ^40^, and that fine-tuning for calibration specifically yields measurable improvements even with constrained training sets ^41^. While larger datasets may yield further improvements, the statistically significant gains observed here confirm that targeted metacognitive supervision is effective at this scale. Expanding the training corpus remains a direction for future work.

The evaluation datasets were then carefully chosen to test the generalisation and robustness of this learned skill, categorised as follows:

- *Broad-domain reasoning*: To assess general performance on complex, multi-task problems, we used established benchmarks like MMLU^42^, ARC, and HellaSwag.
- *Out-of-Domain (OOD) generalisation test*: This is the most critical test of our hypothesis. We used datasets intentionally dissimilar to our training data, such as CommonsenseQA^43^ and a specific slice of Natural Questions, to determine if the metacognitive capabilities truly transfers as a generalisable skill rather than being a memorised pattern.
- *Specialised reliability test*: To evaluate a crucial real-world application of metacognition–detecting misinformation–we used TruthfulQA^44^ and our custom-built RAGTruth dataset. These test the model’s ability to express uncertainty when faced with potentially misleading or false information.

Our evaluation framework was designed to provide a holistic assessment of model performance, moving beyond simple accuracy to capture the nuanced dimensions of metacognitive capabilities. To facilitate detailed analysis, particularly for metrics like over/under-confidence ratios, we categorise the model’s continuous confidence scores into discrete bins (e.g., low [0-0.4], medium [0.4-0.7], high [0.7-1.0]). The key metrics employed are detailed in Table 1.

**Table 1:** A multi-faceted framework for evaluating model performance and metacognitive capabilities.

Metric group	Metric	Purpose and significance in this study
Task performance	Accuracy	Measures the fundamental ability of the model to perform the task correctly. Serves as a baseline to ensure metacognitive enhancements do not come at a significant cost to core competence.
Calibration performance	Brier score	A comprehensive measure of calibration that quantifies the mean squared difference between predicted confidence and actual outcomes. A lower score indicates superior overall reliability^45^.
	Expected calibration error (ECE)	Diagnoses miscalibration by measuring the gap between a model’s stated confidence and its actual accuracy. It is a primary indicator of whether a model’s self-assessment is trustworthy^23^.
	Negative log-likelihood (Log Loss)	Strictly evaluates the quality of a model’s probabilistic predictions, heavily penalising overconfident predictions that turn out to be incorrect.
Metacognitive behaviour	Confidence–accuracy analysis	Uses calibration curves for visual diagnosis, showing the relationship between confidence levels and observed accuracy. An ideal model follows the diagonal line.
	Over/Under-confidence Ratio	Quantifies a model’s systematic bias towards being over- or under-confident, providing insight into the nature of its self-assessment errors.

To ensure the transparency and integrity of our findings, the code for our training and evaluation scripts, along with the LoRA adapter weights for our models, will be made available upon request to support reproducibility.

## Results

The results are organised to first establish the overall superiority of our metacognitive models across a wide spectrum of powerful baselines, followed by a validation of their statistical significance. We then present in-depth analyses of specific metacognitive behaviours and conclude with an ablation study to isolate the impact of our proposed fine-tuning methodology.

Our primary analysis involved a large-scale comparison against a diverse set of models, including widely-used open-source foundation models (Llama-3-8B, Phi-3-Mini, Mistral-7B, Gemma-7B, Qwen2-7B) and SOTA proprietary systems (GPT-4o, Claude-3.5-Sonnet). The overall performance, summarised in Table 2, reveals a clear and consistent pattern. Our Meta-Llama-3-8B and Meta-Phi-3-Mini models not only demonstrate a striking advantage in calibration metrics, but they also show a modest improvement in task accuracy over their respective base models. They achieve the lowest (best) Brier Scores and Expected Calibration Errors (ECE), confirming their superior ability to realistically assess their own response accuracy compared to all other models.

**Table 2:** Comprehensive performance comparison across all models.

Category	Model	Accuracy	Brier score	ECE
Metacognitive model	Llama-3-8B-Instruct	0.853	0.122	0.224
Base model	Llama-3-8B-Instruct	0.837	0.138	0.252
Metacognitive model	Phi-3-Mini-4k-Instruct	0.847	0.128	0.231
Base model	Phi-3-Mini-4k-Instruct	0.820	0.155	0.274
Base model	Mistral-7B-Instruct-v0.3	0.827	0.147	0.264
Base model	Gemma-7B	0.803	0.169	0.291
Base model	Qwen2-7B	0.840	0.135	0.246
SOTA	GPT-4o	0.870	0.209	0.234
SOTA	Claude-3.5-Sonnet	0.877	0.166	0.208

Notably, GPT-4o returns a comparatively higher Brier Score (0.209) than several smaller models, including our metacognitive models. This is attributable to the manner in which proprietary models such as GPT-4o express confidence: rather than outputting calibrated probabilistic estimates, they tend to verbalise confidence as natural language expressions that are subsequently mapped to numerical scores. This extraction process introduces systematic bias, as such models are known to exhibit a tendency towards overconfident verbal expressions of certainty ^5^, which inflates the Brier Score independently of task accuracy. Consequently, the Brier Score for SOTA models should be interpreted with this methodological caveat in mind, rather than as a direct indication of inferior calibration relative to smaller open-source models. Statistical testing confirmed that these improvements were not attributable to chance. The reduction in Brier Score for our metacognitive models compared to their respective base model counterparts was found to be statistically significant for both architectures (Llama: *p=0.023*, Cohen’s *d=1.456*; Phi: *p=0.031*, Cohen’s *d=1.247*), confirming that the improvements are not attributable to chance. This provides strong evidence that the Predictive Metacognition framework induces a genuine and measurable enhancement of model reliability. Beyond these aggregate scores, we performed in-depth analyses of the models’ metacognitive behaviour. To contextualise these results against established post-hoc calibration baselines, Table 3 compares our approach with Temperature Scaling ^23^ and Conformal Prediction ^25^ on representative models. Temperature Scaling and Conformal Prediction operate at inference time on frozen models, whereas our framework modifies the training objective to achieve intrinsic calibration. As shown in Table 3, while Temperature Scaling yields improvements for SOTA models such as GPT-4o and Claude-3.5-Sonnet, it does not address the underlying metacognitive deficit in base models. Our training-time approach, by contrast, achieves calibration improvements that persist without any post-hoc adjustment, representing a more principled and scalable pathway to reliable self-assessment.

**Table 3:** Comparison of calibration methods across representative models.

Category	Model	Original		After Temp. scaling		CP
		Brier	ECE	Brier	ECE	Coverage
Metacognitive	Llama-3-8B-Instruct	0.122	0.224	*intrinsic calibration*		–
Base	Llama-3-8B-Instruct	0.138	0.252	0.211	0.107	0.996
Metacognitive	Phi-3-Mini-4k-Instruct	0.128	0.231	*intrinsic calibration*		–
Base	Phi-3-Mini-4k-Instruct	0.155	0.274	0.250	0.185	1.000
SOTA	GPT-4o	0.209	0.234	0.127	0.051	0.982
SOTA	Claude-3.5-Sonnet	0.166	0.208	0.016	0.008	0.976

Temperature Scaling (TS) and Conformal Prediction (CP) are post-hoc inference-time baselines applied to frozen models, whereas our Predictive Metacognition framework modifies the training objective to achieve intrinsic calibration. TS Brier and TS ECE denote scores after temperature optimization on a held-out calibration set (*n=250*). CP Coverage reports empirical coverage at target level *1-α = 0.90*. Temperature Scaling and Conformal Prediction operate at inference time without modifying the learning objective, whereas our framework integrates metacognitive supervision during training.

**Fig. 3:**
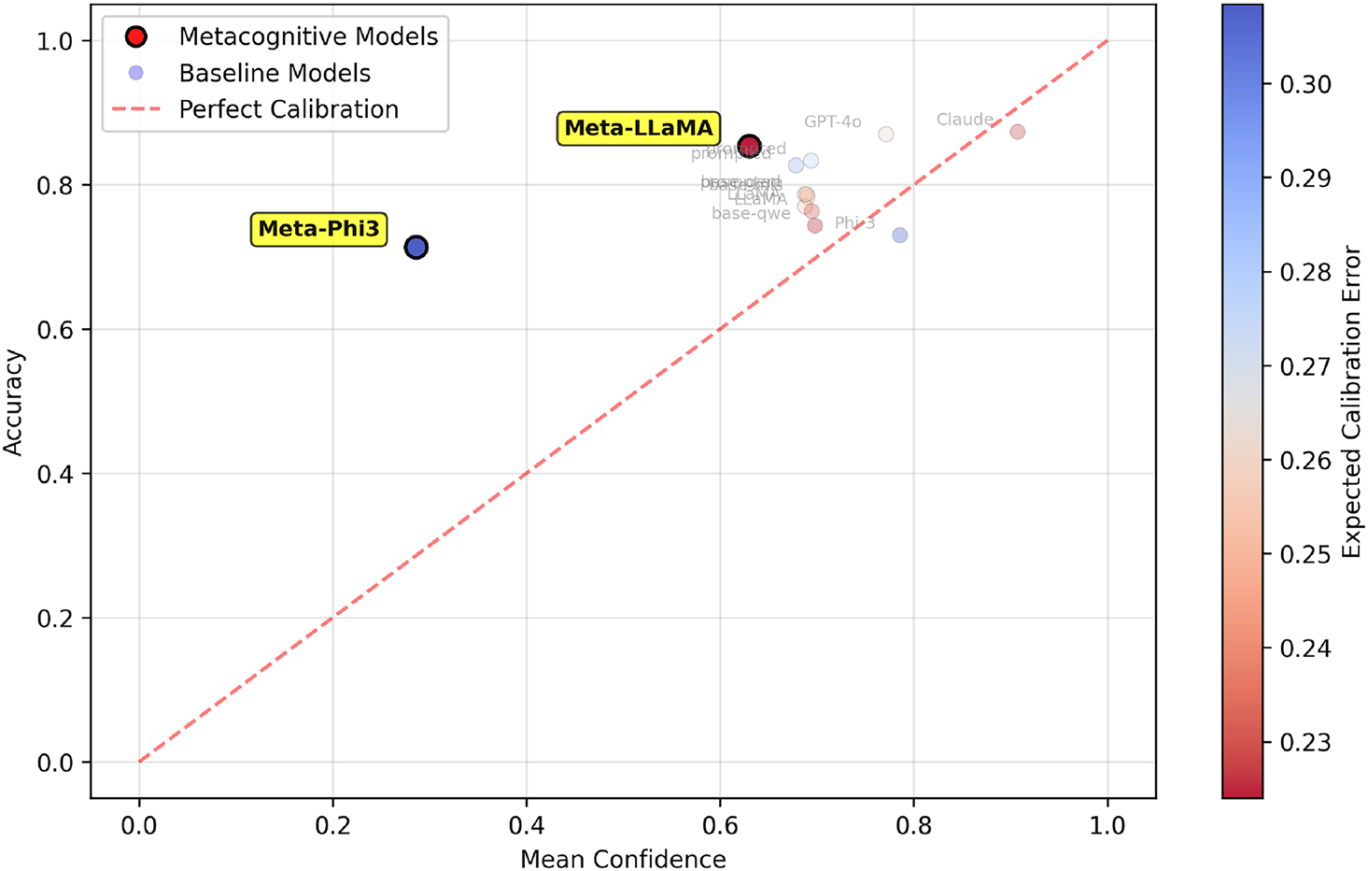
Confidence vs. accuracy calibration for representative models. Colour indicates expected calibration error (ECE).

Figure  3 visualises the confidence calibration of representative models. Each point represents a model’s mean confidence plotted against its mean accuracy; points lying below the diagonal indicate overconfidence, while points above indicate under-confidence. Metacognitive-Llama lies closest to the ideal diagonal among all models shown, in stark contrast to its base version and the SOTA competitors, which both exhibit significant overconfidence. This provides intuitive evidence of our model’s superior calibration. The critical test of our framework, however, was its ability to generalise this skill.

**Fig. 4:**
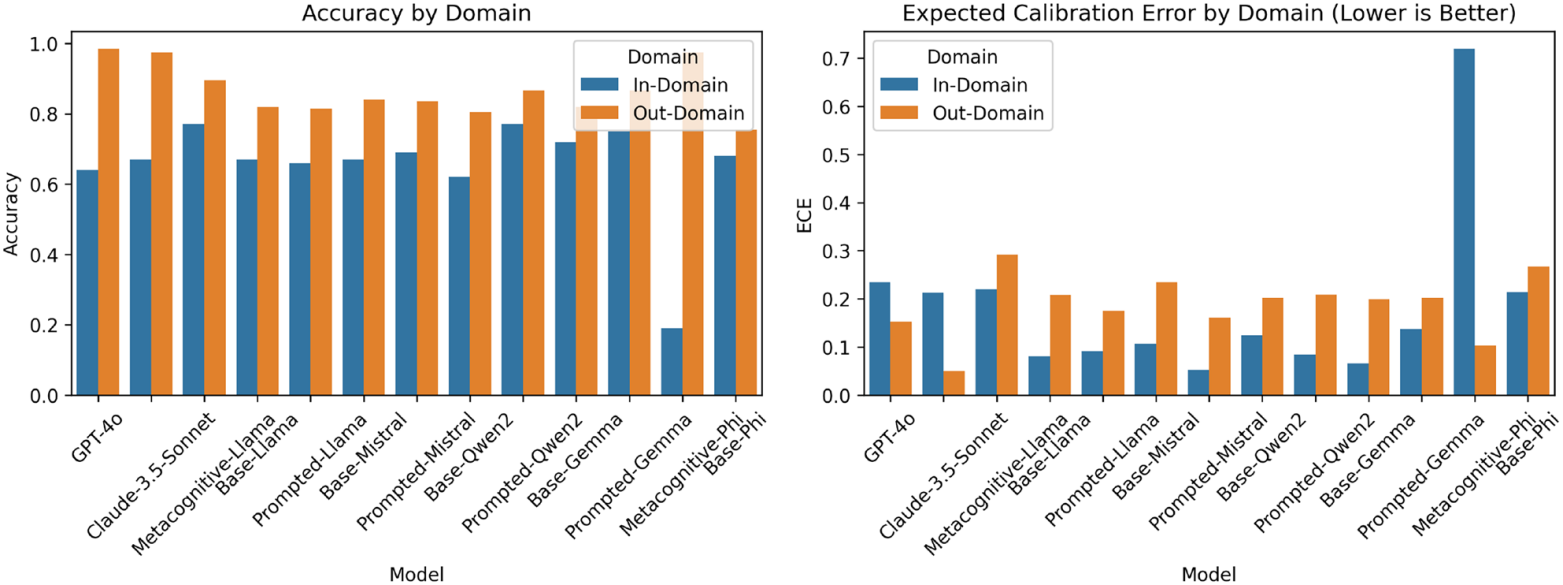
Comparison of model calibration on in-domain (TruthfulQA) and out-of-domain (CommonsenseQA) datasets.

We hypothesised that if metacognitive fine-tuning had instilled a generalisable self-monitoring skill—rather than mere pattern memorisation from training data—models should respond to out-of-domain tasks with appropriately increased uncertainty, reflected in higher ECE relative to the in-domain condition. Figure  4 confirms this hypothesis. As shown in the left panel, all models exhibit higher task accuracy on the OOD dataset (CommonsenseQA) than on the in-domain dataset (TruthfulQA). This is attributable to the nature of the two datasets: TruthfulQA is specifically designed to elicit false beliefs and test resistance to misleading information, making it inherently more challenging, while CommonsenseQA relies on everyday commonsense reasoning where surface-level patterns may be more readily exploited. Critically, however, as shown in the right panel, ECE degrades under domain shift for all models, confirming that the domain shift introduces genuine uncertainty. The key finding is that our metacognitive models show comparatively smaller ECE degradation relative to baseline models, demonstrating a more appropriate and honest response to operating in an unfamiliar domain—strong evidence that the learned metacognitive skill generalises beyond the training distribution. To further quantify these behavioural differences, we analysed their confidence patterns, as shown in Table 4.

**Table 4:** Quantitative analysis of metacognitive behaviours.

	Metacognitive Model	Base Model	SOTA (GPT-4o)
Mean confidence	0.630	0.638	0.876
Overconfidence	0.285	0.303	0.942
Under-confidence	0.310	0.310	0.153
Metacognitive awareness	0.777	0.754	0.814

Both the metacognitive model and base model are based on Llama-3-8B-Instruct. *Metacognitive Awareness* is defined as the point-biserial correlation between the model’s expressed confidence scores and binary correctness labels ($r_{pb}$), quantifying the degree to which confidence aligns with actual performance. Higher values indicate better self-assessment calibration

The results are revealing. Baseline and SOTA models exhibit a strong tendency towards overconfidence. In contrast, Metacognitive-Llama significantly curbs this tendency, showing a more balanced and cautious confidence profile. This is reflected in its superior Metacognitive Awareness score, indicating that its confidence assessments are more aligned with its actual performance.

Finally, to isolate the source of these performance gains, we conducted an ablation study. The results, shown in Table 5, deconstruct the performance improvements by comparing the base model, a prompt-engineered version, and our final metacognitive model.

**Table 5:** Ablation study of metacognitive finetuning on Llama-3-8B-Instruct.

Metric	Base model	Prompted model	Metacognitive model
Accuracy	0.837	0.843	0.853
Brier score	0.138	0.132	0.122
Expected calibration error (ECE)	0.252	0.243	0.224

The data clearly shows that while prompting provides a modest benefit, the most significant leap in calibration performance is directly attributable to our fine-tuning methodology. This confirms that the proposed learning framework, not just task-specific instructions, is the key driver of the enhanced metacognitive capabilities.

## Discussion

The empirical results presented in the preceding chapter provide strong support for our central hypothesis: fine-tuning large language models using the Predictive Metacognition framework leads to a significant and robust improvement in their self-assessment capabilities, particularly in confidence calibration, without substantially degrading core task performance. This was demonstrated consistently across multiple model architectures and against SOTA baselines. This chapter aims to interpret these findings, connect them back to the neurobiological principles that motivated this work, critically evaluate the study’s contributions and limitations, and discuss its broader implications for the field of AI.

Our results indicate a fundamental shift in model behaviour. The marked reductions in Brier Score and ECE are more than mere numerical improvements; they represent the formation of a more refined internal model of uncertainty. This suggests a foundational step towards more reliable intrinsic self-monitoring, where the model does not only compute an answer but also generates a calibrated assessment of its own epistemic state in relation to that answer. The significance of this achievement is underscored by the OOD generalisation results. The ability of our metacognitive models to express appropriate uncertainty when faced with unfamiliar tasks is strong evidence that they have learned a generalisable skill of metacognition, rather than merely overfitting to patterns within the training data. This behavioural evolution, from a confidently incorrect system to a more cautious and self-aware agent, is a crucial qualitative leap for building trustworthy AI.

A central tenet of this paper is the bridge between high-level neurobiological theory and practical AI implementation. The following table provides a critical comparison between the inspirational principles and our computational framework, highlighting both the successes of the analogy and its necessary simplifications.

**Table 6:** Comparison of neurobiological principles and their AI implementation.

Neurobiological principle	Analogue in AI implementation (Similarity)	Key differences & Simplifications (Limitation)
*Error-driven learning*: The brain learns by minimising the mismatch (prediction error) between expectations and outcomes, a process modulated by complex neurochemical signals.	*Metacognitive loss function*($L_{meta}$): Directly operationalises this principle by minimising the Binary Cross-Entropy loss between predicted confidence ($\hat{c}$) and the actual outcome ($y_{meta}$), treating this as a computable metacognitive prediction error.	*Binary error signal*: The brain’s graded and complex reward signals are simplified into a binary ’correct/incorrect’ label. The AI implementation does not capture the degree of correctness or error.
*Performance monitoring (ACC)*: The Anterior Cingulate Cortex (ACC) acts as a specialised module for detecting conflict and signalling the need for cognitive adjustment.	*Metacognitive prediction module*: A functional, rather than anatomical, analogue. The confidence prediction head and the custom training loop collectively serve to detect conflict between predicted and actual performance and use it to drive learning.	*Non-modular & functional approximation*: The monitoring function is distributed across trained network weights, not localised in a distinct module. The mechanism is purely computational, lacking the rich neurochemical context of the biological ACC.
*Hierarchical prediction*: The cortex processes information hierarchically, with predictions and errors flowing between levels, from low-level features to high-level concepts.	*Transformer architecture + final-layer supervision*: The inherent layer-stacking of Transformers provides a structural hierarchy. We add a single, high-level supervisory signal at the final stage to predict overall response correctness.	*Shallow metacognitive hierarchy*: The implementation only provides explicit metacognitive supervision at the very top layer. It lacks the deep, recursive feedback loops between layers envisioned by full predictive coding theories.
*Selective attention*: Humans focus cognitive resources on salient information to inform judgments and confidence, a process involving the interaction of multiple brain regions.	*Attention-based confidence estimation*: The model implicitly learns to weigh the importance of its own internal representations (token embeddings, attention patterns) when computing a final confidence score, functionally mimicking a focus on salient reasoning steps.	*Mechanistic attention*: The self-attention mechanism, while powerful, is a mathematical operation for calculating token relevance. It is a highly simplified, mechanistic model of the brain’s dynamic and complex attentional system.

As Table 6 elucidates, our framework represents a deliberate and pragmatic translation of these principles. The simplifications–such as the binary error signal and the shallow supervisory hierarchy–were necessary choices to ensure computational tractability within existing deep learning paradigms and to provide a clear, unambiguous learning signal for the model. Furthermore, our framework’s focus on the functional role of error monitoring, inspired by the ACC, is itself a simplification, as evidence suggests that human metacognition relies on a distributed brain network in which regions like the posterior parietal cortex also play a key role^46^.

This research offers several broader implications. It presents a new direction for AI safety, suggesting a path toward building models with intrinsic self-discipline, as a complement or alternative to external, post-hoc verification systems like Retrieval Augmented Generation (RAG). By demonstrating the functional benefits of neuro-inspired architectural principles, this work also contributes to computational neuroscience, offering a testable model of how metacognitive functions might be realised in neural circuits. The practical applications are significant, particularly in high-stakes domains such as medicine and law, where an AI that reliably knows what it does not know is profoundly more valuable than one that is perpetually overconfident. This approach, which fosters intrinsic self-regulation, aligns with the broader vision of ‘autonomous deep learning’ where systems can operate reliably without constant human supervision^47^. The importance of reliable prediction and interpretable explanation in high-stakes AI applications extends beyond language models. Recent work in computational biology has demonstrated that producing trustworthy predictions requires not only accurate outputs but also transparent explanations of the underlying decision process–for instance, through graphlet-based explanation generators for graph neural networks applied to biological interaction networks ^48^ and diffusion-based graph attention networks for drug response prediction ^49^. Our work contributes to this broader agenda by addressing the analogous challenge in LLMs: ensuring that confidence estimates are not merely accurate but also reliably interpretable as signals of epistemic state. This work also contributes to a growing stream of AI safety research concerned with quantifying discrepancies between what LLMs express and what they enact. Recent work has demonstrated that LLMs may exhibit systematic divergences between stated preferences—the values they endorse when prompted—and revealed preferences—the choices they make in contextualised scenarios^50,51^. Furthermore, value-based approaches to identifying AI risk have shown that measuring an LLM’s implicit value prioritisation can serve as an early warning system for potentially unsafe behaviours^52^. Our framework complements these efforts by addressing a related but distinct challenge: not merely what values an LLM endorses, but whether its expressed confidence reliably reflects its actual epistemic state.

Our study, however, is not without limitations, and these naturally point toward rich avenues for future research. The simplifications noted in Table 6 form a clear research agenda. Future work could explore more sophisticated learning signals beyond binary labels — for instance, graded confidence supervision that assigns partial correctness scores rather than a single binary label–potentially drawing on techniques from reinforcement learning to capture the nuanced degree of correctness in model responses. A deeper investigation into implementing true hierarchical monitoring, with metacognitive feedback loops integrated at multiple layers of the Transformer architecture, could yield further improvements. Furthermore, a key direction for future work is to investigate the scalability of our framework. While this study demonstrates clear efficacy on models up to 8 billion parameters, validating how these metacognitive improvements scale on models with tens to hundreds of billions of parameters is a critical next step to confirm the universality of our approach. Finally, extending this framework of predictive self-monitoring to more complex, autonomous agents could empower them to make more robust and safer decisions when planning, reasoning, and using tools. This path forward resonates with prior work in Neural Networks on developing cognitive agents with dedicated metacognitive architectures^53,54^, suggesting a fruitful convergence of our bottom-up approach with top-down agent design.

## Conclusion

Modern LLMs, despite their remarkable capabilities, are hindered by a critical and pervasive reliability crisis. This crisis stems from a fundamental deficit in metacognition–an inability to realistically assess their own knowledge and express calibrated confidence, leading to the persistent issues of hallucination and unwarranted overconfidence. This work confronted this challenge directly by moving beyond reactive, external fixes and targeting the model’s intrinsic processing.

We proposed and implemented Predictive Metacognition, a novel framework for endowing LLMs with an internal self-monitoring capability. Drawing inspiration from the principles of predictive processing and error monitoring in human cognitive neuroscience, we developed a specialised fine-tuning methodology that trains models to concurrently generate answers and predict the likelihood of their own accuracy. This dual-objective approach reshapes the model’s internal landscape, compelling it to form a more nuanced representation of its own uncertainty.

Our comprehensive experiments yielded clear and statistically significant results. Models trained with the Predictive Metacognition framework consistently demonstrated superior confidence calibration across a wide range of tasks and against a diverse suite of strong baseline and SOTA models. Crucially, they exhibited a more robust and ‘honest’ self-assessment capability when faced with out-of-domain challenges, suggesting that they learned a generalisable metacognitive skill rather than merely memorising patterns.

The primary contribution of this research is the demonstration of a viable path toward building AI systems with intrinsic self-regulation. By successfully translating principles from neuroscience into a computational framework, we have shown that it is possible to create models that are not only highly capable but also more trustworthy and aware of their own limitations. This work represents a paradigm shift, moving the focus of AI safety from external scaffolding to the internal discipline of the model itself.

Ultimately, this research constitutes a crucial step toward a future where AI can be trusted not just for what it knows, but for its wisdom in recognising what it does not. The pursuit of reliably self-monitoring AI that can express calibrated uncertainty is no longer a distant philosophical goal, but an active and achievable engineering and scientific endeavour.

## Data Availability

This study utilised publicly available benchmark datasets. The datasets and their access links are as follows: TruthfulQA: https://github.com/sylinrl/TruthfulQA (also available at https://huggingface.co/datasets/truthful_qa). MMLU (Massive Multitask Language Understanding): https://huggingface.co/datasets/cais/mmlu. ARC (AI2 Reasoning Challenge): https://huggingface.co/datasets/allenai/ai2_arc. HellaSwag: https://huggingface.co/datasets/Rowan/hellaswag. CommonsenseQA: https://huggingface.co/datasets/tau/commonsense_qa. Natural Questions: https://huggingface.co/datasets/google-research-datasets/natural_questions. RAGTruth: https://github.com/ParticleMedia/RAGTruth. The synthetic metacognitive training dataset (4000 examples) and the LoRA adapter weights for our models are available from the corresponding author on reasonable request.

## Code availability

The code for our training and evaluation scripts, along with the LORA adapter weights for our models, will be made available upon request to support reproducibility.
